# An In-Vitro Cell Model of Intracellular Protein Aggregation Provides Insights into RPE Stress Associated with Retinopathy

**DOI:** 10.3390/ijms21186647

**Published:** 2020-09-11

**Authors:** Eloise Keeling, Annabelle J. Culling, David A. Johnston, David S. Chatelet, Anton Page, David A. Tumbarello, Andrew J. Lotery, J. Arjuna Ratnayaka

**Affiliations:** 1 Clinical and Experimental Sciences, Faculty of Medicine, University of Southampton, MP806, Tremona Road, Southampton SO16 6YD, UK; E.E.Keeling@soton.ac.uk (E.K.); ajc1g16@soton.ac.uk (A.J.C.); A.J.Lotery@soton.ac.uk (A.J.L.); 2 Biomedical Imaging Unit, University of Southampton, MP12, Tremona Road, Southampton SO16 6YD, UK; D.A.Johnston@soton.ac.uk (D.A.J.); D.S.Chatelet@soton.ac.uk (D.S.C.); A.Page@soton.ac.uk (A.P.); 3 Biological Sciences, Faculty of Environmental and Life Sciences, Life Sciences Building 85, University of Southampton, Highfield Campus, Southampton SO17 1BJ, UK; D.A.Tumbarello@soton.ac.uk; 4 Eye Unit, University Hospital Southampton NHS Foundation Trust, Southampton SO16 6YD, UK

**Keywords:** RPE, oxidized POS, proteolysis, lysosomes, autophagy, autofluorescence, retina, AMD, diet, aging

## Abstract

Impaired cargo trafficking and the aggregation of intracellular macromolecules are key features of neurodegeneration, and a hallmark of aged as well as diseased retinal pigment epithelial (RPE) cells in the eye. Here, photoreceptor outer segments (POS), which are internalized daily by RPE cells, were modified by UV-irradiation to create oxidatively modified POS (OxPOS). Oxidative modification was quantified by a protein carbonyl content assay. Human ARPE-19 cells were synchronously pulsed with POS or OxPOS to study whether oxidatively modified cargos can recapitulate features of RPE pathology associated with blinding diseases. Confocal immunofluorescence microscopy analysis showed that OxPOS was trafficked to LAMP1, LAMP2 lysosomes and to LC3b autophagy vacuoles. Whilst POS were eventually degraded, OxPOS cargos were sequestered in late compartments. Co-localization of OxPOS was also associated with swollen autolysosomes. Ultrastructural analysis revealed the presence of electron-dense OxPOS aggregates in RPE cells, which appeared to be largely resistant to degradation. Measurement of cellular autofluorescence, using parameters used to assess fundus autofluorescence (FAF) in age-related macular disease (AMD) patients, revealed that OxPOS contributed significantly to a key feature of aged and diseased RPE. This in vitro cell model therefore represents a versatile tool to study disease pathways linked with RPE damage and sight-loss.

## 1. Introduction

The retinal pigment epithelium (RPE) plays a critical role in vision and is intimately associated with photoreceptors of the neuroretina. Defects in the RPE monolayer are linked with common blinding diseases such as age-related macular degeneration (AMD) as well as with many retinal dystrophies [1,2,3,4]. RPE cells are amongst the most phagocytic in the body and specialize in internalizing photoreceptor outer segments (POS) from overlying photoreceptors as part of the daily visual cycle. Each RPE cell is estimated to process up to 4000 disks per day [5]. The incomplete degradation of POS contributes to elevated proteolytic stress, which increases over several decades, adding to the likelihood of disease in later life [6,7]. A characteristic feature of RPE is the accumulation of intracellular lipofuscin, which is thought to result from non-enzymatic reactions of vitamin A aldehyde in photoreceptors, and subsequent transfer to RPE cells in POS [8]. Hence, ~20% of the RPE cytoplasm becomes filled with lipofuscin by the eighth decade of life [9]. Lipofuscin has been demonstrated to possess cytotoxic properties, which can specifically target terminal compartments of the proteolytic pathway [10,11]. The gradual accumulation of intracellular material is a feature of many post-mitotic cells [12,13] and is not exclusive to RPE. However, intense light levels in the retina, particularly in the lower wavelengths [14] alongside high metabolic activity coupled with hypoxic conditions, create a unique environment, where proteins and lipids become susceptible to modification by photo-oxidation. This results in the formation of a variety of intracellular compounds, such as pyridinium bis-retinoid A2E, malondialdehyde (MDA) and 4-hydroxynonenal (HNE), amongst others, which are known to have different pathogenic effects in the RPE [15,16,17,18,19,20,21]. Such compounds also target activities of the proteolytic pathway. The accumulation of these molecules further exacerbates the susceptibility of RPE cells to blue light-induced damage [22,23]. Constituents of long-chain polyunsaturated fatty acids (PUFAs), such as docosahexaenoic acid (DHA), present at high quantities in POS, also contribute to oxidative stress in RPE cells. Moreover, oxidized PUFAs from POS cannot be efficiently degraded in RPE lysosomes, which adds to proteolytic stress [21]. Carboxyethylpyrrole (CEP), generated from oxidative modification of DHA, is used as a serum biomarker of AMD [24]. Approximately a billion photoreceptor disks are internalized over a 70-year period. This, combined with the effects of the aforementioned processes, places an enormous proteolytic burden on the RPE. This burden is considered to be the highest in the human body. A nuanced understanding of these mechanisms could result in elucidating the etiology of retinopathy and its progression, so that effective new treatments can be developed for diseases that currently result in irreversible sight-loss.

This study sets out to elucidate how increased proteolytic stress could influence activities of the phagosome and autophagy-lysosomal pathways of RPE cells. We used an established in vitro RPE cell model [25,26,27] and studied effects of proteolytic stress by utilizing ultraviolet (UV) irradiation to generate oxidatively modified POS, termed OxPOS, which is reported to accumulate within RPE [28,29]. The trafficking of OxPOS cargos to specific intracellular compartments was compared with normal POS in healthy ARPE-19 cells. Data from confocal immunofluorescence studies were analyzed by an automated, unbiased algorithm to obtain a quantitative measure of cargo trafficking. These investigations were supported by ultrastructural studies, which provided additional insights into cargo degradation, revealing molecular events underpinning the contrasting fates of intracellular POS and OxPOS. Since autofluorescence is a characteristic feature of RPE cells which is thought to originate from accumulated intracellular material [30], we studied whether this feature of the aged and diseased retina could also be recapitulated in our model.

## 2. Results

### 2.1. Preparation and Characterization of Photoreceptor Outer Segments (POS) and Modified POS (OxPOS)

Porcine photoreceptor outer segments (POS) were isolated as described previously [26,27,31]. In order to modify POS, a proportion of isolated POS were exposed to a 254 nm wavelength beam for 3 h which delivered 120,000 µJ [32,33]. Use of a commercially available UV cross-linking device allowed a standardized approach to generate oxidatively modified POS (OxPOS), which was subsequently visualized in vitro by transmission electron microscopy (TEM). Ultrastructural analysis of POS and OxPOS preparations in vitro showed the arrangement of their morphology prior to internalization by RPE cells (Supplementary Materials Figure S1). The regular organization of outer segment membranes, characteristic of in situ POS, could be clearly visualized. In contrast, membranes had coalesced into an electron-dense aggregate following UV irradiation. The introduction of carbonyl groups into amino acid residues of proteins is a hallmark of oxidative modification [34]. In order to evaluate this, a carbonyl content assay was performed in POS and OxPOS preparations. Measurements were carried out before POS and OxPOS were fed to cultured RPE, so readouts would not be influenced by cellular effects. Our results show that UV-irradiation had significantly increased oxidative modifications of OxPOS preparations (Figure S1).

### 2.2. An Initial Screen Shows that POS and OxPOS Cargos Are Trafficked Differently within the Endolysosomal and Autophagy Systems of RPE Cells

We and others had previously shown that ARPE-19 cells bind and internalize POS via αVβ5 and MerTK receptor-mediated mechanisms. Following initial passage through phagosomes/endosomes and autophagosomes, internalized POS are eventually trafficked to autolysosomal compartments for degradation [4,27,35]. Our previous work described details of this process, where POS are trafficked to early (LAMP1) and mature (LAMP2) lysosomes to reach a peak after 24 h. Cargos were initially trafficked to LC3b-positive autophagic vacuoles at low levels, but increased significantly after 12 h to reach a peak by 48 h [27]. As the timelines of POS trafficking to specific vesicles has been well-defined, with significant co-localization to late compartments shown to occur no earlier than 24 h, the current study carried out an initial screen to compare OxPOS trafficking from 12 h onwards. However, as OxPOS is known to form electron-dense aggregates (Figure S1) [13,28,29], the current study was extended up to 72 h, to delineate potential differences between POS vs. OxPOS cargos. Moreover, additional time points were incorporated into our initial screen, to reveal any nuanced differences in the way POS and OxPOS cargos may be trafficked and processed in RPE cells.

**Figure 1:**
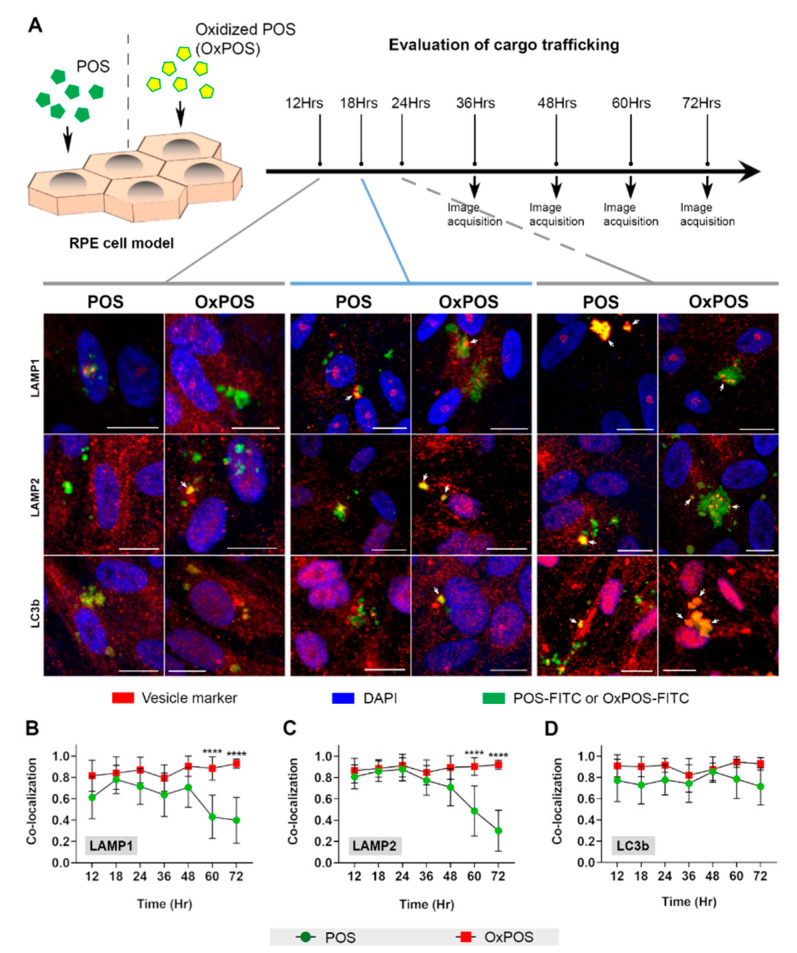
An initial screen evaluating the intracellular cargo trafficking in retinal pigment epithelial (RPE) cells. (**A**) A schematic diagram of the experimental plan, where cultured ARPE-19 cells were fed with either photoreceptor outer segments (POS) or oxidized POS (OxPOS). Trafficking was assessed by confocal immunofluorescence microscopy from 12 h onwards, when cargos are expected to reach late compartments. Following incubation with either POS or OxPOS, a screen spanning the full range of time points in the study was performed. Internalized cargos (green) were observed to co-localize with vesicles in the autophagy-lysosomal pathways (red) and appeared yellow (arrows). Compared to POS, OxPOS appeared to form more noticeable intracellular puncta (indicated by arrows showing prominent/large OxPOS aggregates). Representative images illustrate the contrasting fates of POS vs. OxPOS, which were quantified using an unbiased, automated algorithm described by Costes et al. [36] and shown for (**B**) LAMP1, (**C**) LAMP2 and (**D**) LC3b. At 12 h, POS and OxPOS were co-localized with ≥60% of early and ≥80% mature lysosomes. However, OxPOS continued to be retained at late time points (60 and 72 h), by which time, POS in lysosomes had begun to significantly diminish. In this initial screen, both POS and OxPOS were detected in ≥70% of LC3b-positive vesicles from 12 h onwards, although POS appeared to marginally decline after 60 h. Scale bar in confocal images correspond to 10 µm. The y-axis in graphs B–D show the extent of co-localization where 1.0 = 100% co-localization. Data from 6 images per time point and at least *n* = 10 cells/image. Statistical comparisons using a two-way analysis of variance (ANOVA) followed by Sidak’s multiple comparison test, where a significant difference is indicated as *p* ≤ 0.0001 (****).

Analysis by confocal immunofluorescence microscopy at 12, 18, 24, 36, 48, 60 and 72 h, following the feeding assay, revealed how OxPOS were trafficked compared to POS cargos. POS and OxPOS could be conveniently visualized as they were tagged with fluorescein isothiocyanate (FITC). Assessment of blinded maximum projection images showed no obvious differences between trafficking POS and OxPOS cargos between 12 and 24 h (Figure 1A). Lysosomes and autophagic vacuoles containing both cargo-types displayed a predominantly perinuclear localization. However, compared to POS, OxPOS formed conspicuous intracellular puncta, particularly by 24 h. Data from this initial screen spanning 12–72 h time points were analyzed using an unbiased automated algorithm described by Costes et al. [36], which provided a value for cargo co-localizing with a specific intracellular compartment (Figure 1B–D). Hence, a value of 1.0 on the y-axis represents 100% co-localization at a given time point, which is indicated on the x-axis. Co-localization data showed no obvious differences between POS and OxPOS trafficking to early and mature lysosomes, until late time points, when significant differences began to emerge at 60 and 72 h (Figure 1B,C). No significant differences were observed for either cargo-type in LC3b vesicles in this initial screen (Figure 1D).

### 2.3. Studies Focused on Late Time Points Show That OxPOS Appear Resistant to Degradation and Becomes Sequestered in Lysosomes and Autophagic Vacuoles of RPE Cells

**Figure 2:**
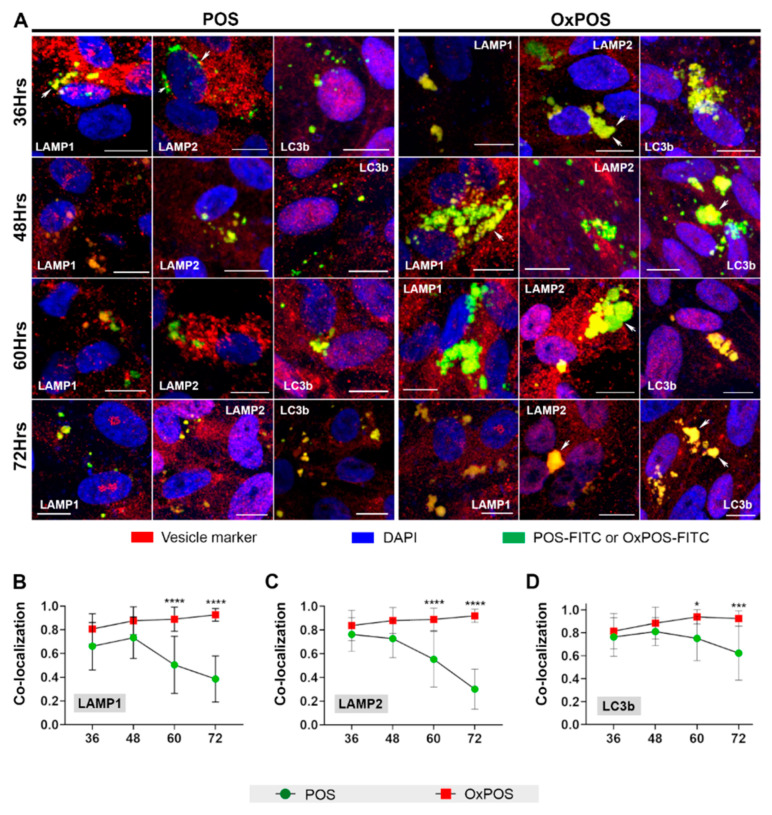
Evaluation of cargo trafficking from 36 to 72 h. RPE cells fed POS or OxPOS were observed trafficked to autophagy-lysosomal compartments. However, noticeable differences between the two cargo-types were not evident until late time points. Hence, additional experiments were carried out focused on 36–72 h. (**A**) Representative confocal images showing co-localization of either POS or OxPOS (green), with specific compartments in the autophagy-lysosomal pathway (red). Areas of co-localization appear yellow (arrows). Whilst the presence of POS declined over time, RPE cells fed with OxPOS showed an accumulation of these cargos. The extent of co-localization was quantified using an unbiased automated algorithm described by Costes et al. [36], with data from the initial screen combined and shown for (**B**) LAMP1, (**C**) LAMP2 and (**D**) LC3b. OxPOS were present in a large proportion of early and mature lysosomes as well as autophagic vacuoles (≥80%), which persisted without significant change throughout the study time course. Scale bar in confocal images correspond to 10 µm. The y-axis in graphs B–D show the extent of co-localization, where 1.0 = 100% co-localization. Data from a minimum of 9 images per time point and at least *n* = 10 cells/images. Statistical comparisons using a two-way ANOVA followed by Sidak’s multiple comparison test, where significant differences are indicated as *p* ≤ 0.05 (*), *p* ≤ 0.001 (***) and *p* ≤ 0.0001 (****).

As our initial screen showed no differences between trafficked POS vs. OxPOS cargos before 48 h, we decided to carry out further experiments focused only on later time points. Hence, in subsequent studies, we selected 36 h after POS or OxPOS exposure as the first time point, followed by 48, 60 and 72 h time points. Assessment of blinded maximum projection images revealed marked differences between POS vs. OxPOS cargos at these times (Figure 2A). POS cargos co-localized with lysosomes and autophagic vacuoles in a predominantly perinuclear fashion. By contrast, OxPOS appeared as large aggregates, which were primarily distributed in a perinuclear manner, but also seen in other areas of the cell. OxPOS appeared to persist without any obvious change until 72 h (the final time point after initial exposure to RPE cells), when POS in LAMP and LC3b compartments appeared to diminish. The results from these assays were analyzed using the Costes method as before [36], and combined with data from our initial screen, to provide a comprehensive overview revealing divergent intracellular fates of POS vs. OxPOS cargos in RPE cells (Table S1).

The extent of POS co-localizing with early lysosomes increased from 12 h onwards and remained largely stable (from 78% at 18 h to 73% until 48 h), after which they diminished rapidly. POS trafficking to mature lysosomes followed a similar pattern, where cargos reached a peak at an earlier time point of 24 h (88% of LAMP2 vesicles were positive for POS) to diminish thereafter. Most LC3b-positive compartments contained POS (77% at 12 h to 75% by 60 h), which started to diminish at 72 h (Table S1). Our data indicate that POS remained in autolysosomes of healthy RPE cells until 72 h, at least in some fashion, and were not as rapidly degraded as has been thought before.

Parallel studies carried out for OxPOS revealed strikingly different outcomes. OxPOS cargos also co-localized with LAMP1 vesicles from 12 h onwards, but at consistently higher levels compared to POS, until 60 h (Table S1). At the 60 h time point, 89% of LAMP1 vesicles were positive for OxPOS, whilst only 50% contained POS. At the final time point of 72 h, 93% of early lysosomes contained OxPOS, whereas POS cargos had diminished to 39% (Figure 2B). The behavior of OxPOS in LAMP2 compartments showed a similar pattern where cargos were associated with mature lysosomes from 12 h onwards, but at constantly higher levels compared to POS. However, there were no significant differences between the two cargo-types until 60 h, when 89% of LAMP2 vesicles contained OxPOS compared to only 55% for POS (Table S1). By combing results from our initial screen (Figure 1) with follow-up studies focused only on late time points (36–72 h), we increased the power of our data (which is shown in Figure 2). Combined data revealed that by 72 h, 92% of mature lysosomes contained OxPOS, compared to only 30% for POS (Figure 2C). Both POS and OxPOS were trafficked to LC3b-positive vesicles, with the latter showing a higher extent of co-localization from 12 h onwards. Significant differences became evident from 60 h onwards, when data from our initial screen was combined with data from experiments focused on late time points (Table S1). Hence, at 60 h, 94% of autophagic vacuoles contained OxPOS compared to only 75% for POS. Values at the 72 h time point were 92% for OxPOS and 62% for POS respectively (Table S1 and Figure 2D), indicating that POS had begun to be degraded, whereas OxPOS remained sequestered without any obvious signs of breakdown. These findings were supported by the accumulation of large intracellular aggregates observed in OxPOS-treated cells.

### 2.4. Co-Localization of OxPOS Cargos to Autophagosomes and Lysosomes Results in Enlarged Vesicles

**Figure 3:**
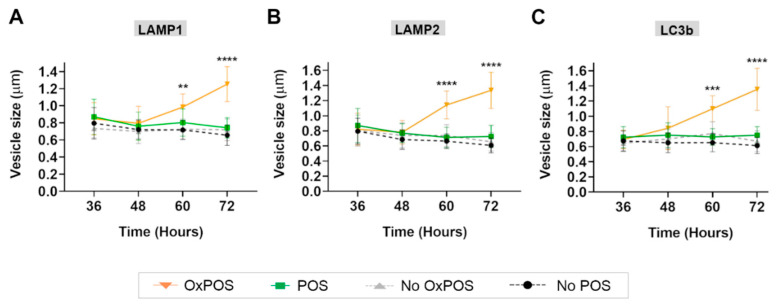
The effects of different cargo-types on the size of autophagy-lysosomes in RPE cells. The diameter of vesicles with and without POS or OxPOS was quantified in blinded confocal images at late time points and shown for (**A**) LAMP1, (**B**) LAMP2 and (**C**) LC3b. Significant differences were observed showing OxPOS associated with enlarged early and mature lysosomes at 60–72 h, compared to equivalent compartments without cargos in the same culture. Similarly, LC3b bodies trafficking OxPOS were also swollen by 60–72 h compared to autophagy vacuoles without these cargos in the same culture. The extent of swelling in OxPOS-carrying vesicles was considerable, which significantly increased in diameter compared to vesicles carrying POS or those without any cargos. Data from a minimum of 5 measurements from each of 9 fields of view and at least *n* = 10 cells/field per time point. Statistical comparisons using a two-way ANOVA followed by Tukey’s multiple comparison test, where significant differences are indicated as *p* ≤ 0.01 (**), *p* ≤ 0.001 (***) and *p* ≤ 0.0001 (****).

Studies of confocal images indicated that OxPOS cargos were associated with enlarged vesicles. To quantify this phenomenon, the diameter of LAMP1, LAMP2 and LC3b compartments with and without POS or OxPOS cargos was measured on single plane images from the middle of a confocal z-stack. The investigator was blinded to the identity of the images. Studies were focused on late time points (36 h onwards) when differences began to emerge between the two cargo-types. Co-localization of POS to lysosomes and autophagy bodies did not result in a significant change to vesicle diameter at any of the time points (POS vs. No POS data: Table S2). The relative sizes of early and mature lysosomes as well as autophagic vacuoles were also consistent with reported organelle dimensions in terminal stages of the proteolytic pathway [4,27]. Co-localization of OxPOS to LAMP1 compartments had no initial effect until 60 h, when the vesicle diameter increased from 0.73 ± 0.12 µm (without cargo) to 0.98 ± 0.15 µm (with OxPOS). By 72 h, the size of LAMP1 vesicles with OxPOS cargos had further increased to 1.26 ± 0.2 µm. Similar effects were recorded in LAMP2 vesicles at 60 h, when the presence of OxPOS resulted in the diameter of mature lysosomes to increase from 0.74 ± 0.14 µm (no cargo) to 1.14 ± 0.12 µm, with a further increase at 72 h to 1.34 ± 0.24 µm (Table S2). OxPOS trafficking to autophagy bodies also resulted in a significant increase in vesicle diameter at 60 h from 0.77 ± 0.15 µm (no cargo) to 1.01 ± 0.17 µm (with OxPOS). This increased further to 1.36 ± 0.28 µm by 72 h when the size of autophagic vacuoles without any cargo (0.61 ± 0.11 µm in No POS and 0.67 ± 0.14 µm in No OxPOS cultures) or with POS (0.75 ± 0.11 µm) remained unchanged (Table S2). The summary graphs show the statistical significance between POS vs. OxPOS in LAMP1 (Figure 3A), LAMP2 (Figure 3B) and LC3b compartments as a function of time (Figure 3C).

### 2.5. Ultrastructural Analysis Revealed Molecular Insights into POS and OxPOS Cargos within RPE Cells

**Figure 4:**
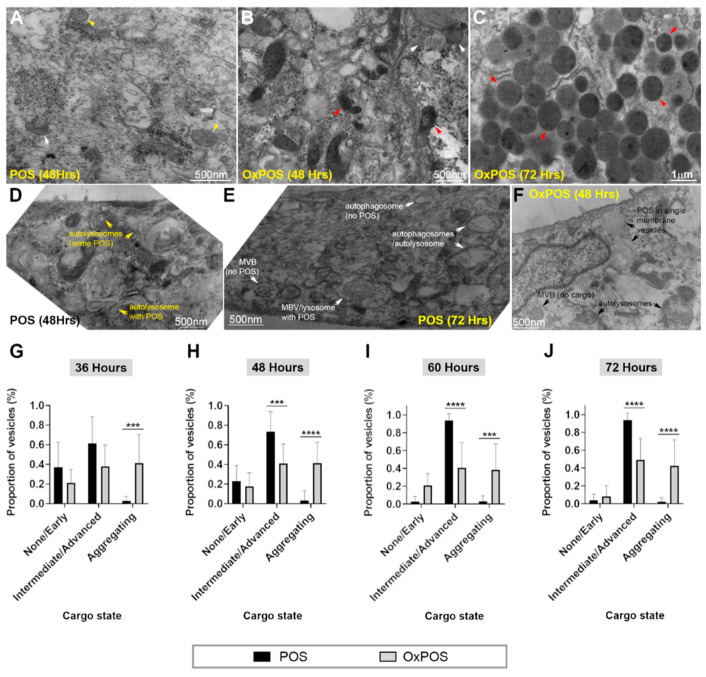
Ultrastructural evaluation of cargos within late compartments of RPE cells. The state of internalized cargos was assessed following exposure of cultured RPE cells to either POS or OxPOS. Morphological assessment revealed trafficked cargos that (1) showed no indication of breakdown or early degradation, (2) intermediate or advanced stages of breakdown or (3) displayed an aggregating phenotype. (**A**) A representative electron micrograph after 48 h, which show POS with characteristic fingerprint-like morphology and without obvious evidence of any degradation (white arrow). On occasion, vesicles containing uniform granular material were also observed (yellow arrows), presumably from endogenous content. (**B**) A representative electron micrograph of cells fed with OxPOS after 48 h shows some evidence of intermediate or advanced stages of cargo breakdown (white arrows), however a majority appear as electron-dense material (red arrows). (**C**) 72 h after cultured RPE cells were initially exposed to OxPOS, a significant proportion of vesicles appeared to contain cargos with a largely homogenous electron-dense morphology. (**D**,**E**) Representative electron micrographs showing POS cargos at 48 and 72 h in a variety of late compartments, including multivesicular bodies (MVBs) and autophagosomes/autolysosome-like structures. POS could be visualized in varying stages of degradation, whilst some compartments have no discernable cargo. (**F**) A representative electron micrograph showing evidence of OxPOS degradation at 48 h. Scale bars in A, B, D–F correspond to 500 nm, whilst scale bar in C corresponds to 1 µm. Trafficked cargos in blinded electron micrographs were grouped as described before, quantified and shown for (**G**) 36 h, (**H**) 48 h, (**I**) 60 h and (**J**) 72 h. As anticipated, the extent of POS breakdown increased over time. Although some OxPOS appeared to be degraded, a significant proportion formed conspicuous intracellular aggregates with a homogenous electron-dense morphology. These were present from the 36 h time point and persisted without obvious evidence of any degradation throughout the time course. For ease of categorizing, vesicles with endogenous material (yellow arrow in A) were grouped into the same category as aggregating OxPOS (red arrows in B–C). This method however, made no significant impact on the large number of aggregating OxPOS quantified in RPE cells. Data from a minimum of *n* = 10 fields/per time point. Statistical comparisons using a two-way ANOVA followed by Sidak’s multiple comparisons test, where significant differences are indicated as *p* ≤ 0.001 (***) and *p* ≤ 0.0001 (****).

TEM studies were carried out to support findings from confocal immunofluorescence experiments, and to evaluate the extent of cargo degradation within intracellular compartments of RPE cells as a function of time. Examination of blinded electron micrographs showed that cargos were either in the process of being degraded or alternatively, formed intracellular aggregates. The former were graded according to the extent of cargo breakdown: (1) No obvious breakdown or early indication of degradation, and (2) intermediate or advanced stages of breakdown. Internalized POS could be identified by their characteristic features, where outer segment membranes were arranged in concentric rings, consistent with their reported morphology [37,38]. Cargos with this morphology and without any obvious signs of degradation were assigned to group 1. Vesicles with cargos in various stages of degradation, evidenced by electron-dense debris within compartments, were categorized as group 2. OxPOS also showed some indication of degradation. However, most OxPOS formed a uniform electron-dense morphology (Figure 4B,C), which was classed as aggregating. Occasionally, in RPE cells fed with POS or OxPOS, we also observed vesicles with uniformly distributed granular content (Figure 4A). We reasoned that these may have originated from endogenous material, particularly as experiments used RPE cells that had been in culture for several months [25,26]. These appeared to be few in number and could not always be reliably distinguished from aggregating OxPOS and were therefore grouped into the same category. Examples of the aforementioned POS and OxPOS organelles are shown in representative electron micrographs (Figure 4A–F). The relative distribution of cargo in each category was evaluated as a function of time, when differences could be identified between trafficked POS and OxPOS at late time points (Figure 4G–J). At 36 h, no differences were observed, other than evidence of aggregation in OxPOS-fed cells. However, from 48 h onwards, significant differences were recorded between POS and OxPOS cargos in intermediate/advanced stages of degradation and displaying an aggregated phenotype. Collectively, these results indicate that POS cargos were progressively degraded over time, compared to OxPOS. The latter also showed some evidence of breakdown. However, a large proportion of OxPOS formed intracellular aggregates, which remained stable over time and accumulated in late-stage compartments.

### 2.6. Quantification of Luminal Electron Density within Trafficking Vesicles Showed POS Degradation and Formation of Stable OxPOS Aggregates

**Figure 5:**
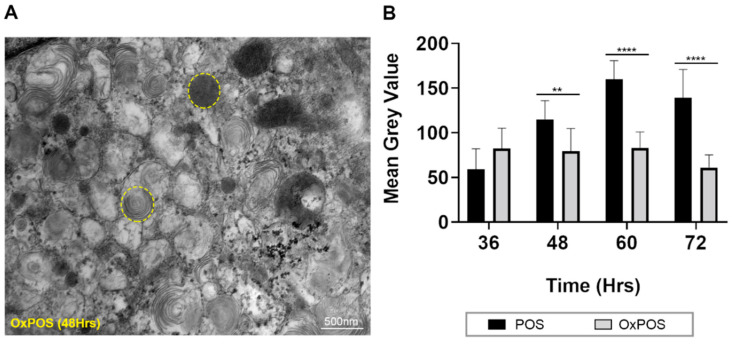
Quantification of cargo content within late compartments as a function of time. To assess whether POS or OxPOS cargos were being degraded or not, trafficking vesicles were demarcated as regions of interest (ROI), and their luminal content quantified by measuring the mean greyscale value. A higher electron density is represented by a lower greyscale value. (**A**) A representative electron micrograph from RPE cells exposed to OxPOS after 48 h. Example ROIs in micrograph show vesicles with distinct cargos (dashed yellow circles). Scale bar corresponds to 500 nm. (**B**) The cargo content in trafficking vesicles were quantified in blinded electron micrographs, which revealed increasing greyscale values within ROIs of POS-trafficking vesicles. By contrast, ROIs with OxPOS showed no appreciable change throughout the time course. Data from a minimum of *n* = 10 fields/per time point. Statistical comparisons using a two-way ANOVA followed by Sidak’s multiple comparison test, where significant differences are indicated as *p* ≤ 0.01 (**) and *p* ≤ 0.0001 (****).

The luminal content of trafficking vesicles was evaluated in order to better understand the contrasting fates of POS and OxPOS cargos. Regions of interest (ROIs) were marked and luminal contents quantified by measuring the greyscale value. A higher electron density is represented by a lower greyscale value. A representative electron micrograph is shown with sample ROIs (Figure 5A). The assessor was blinded to the identity of the TEM micrographs and data obtained without any reference to previous groups and based solely on greyscale values (Figure 5B). No differences between POS vs. OxPOS cargos were observed at 36 h after the feeding assay. However, significant differences in greyscale values between these two cargo-types were recorded at 48, 60 and 72 h. The processing of POS cargos resulted in the gradual breakdown of outer segment membranes and diminished luminal content, which was reflected by elevated greyscale values with increasing time. By contrast, the greyscale values of OxPOS remained largely stable throughout the study, indicating that once formed into a uniform electron-dense morphology, intracellular OxPOS was resistant to proteolytic degradation for at least 72 h following the feeding assay.

### 2.7. POS Degradation and Aggregated OxPOS Are Associated with Deeper Cell Layers as a Function of Time

**Figure 6:**
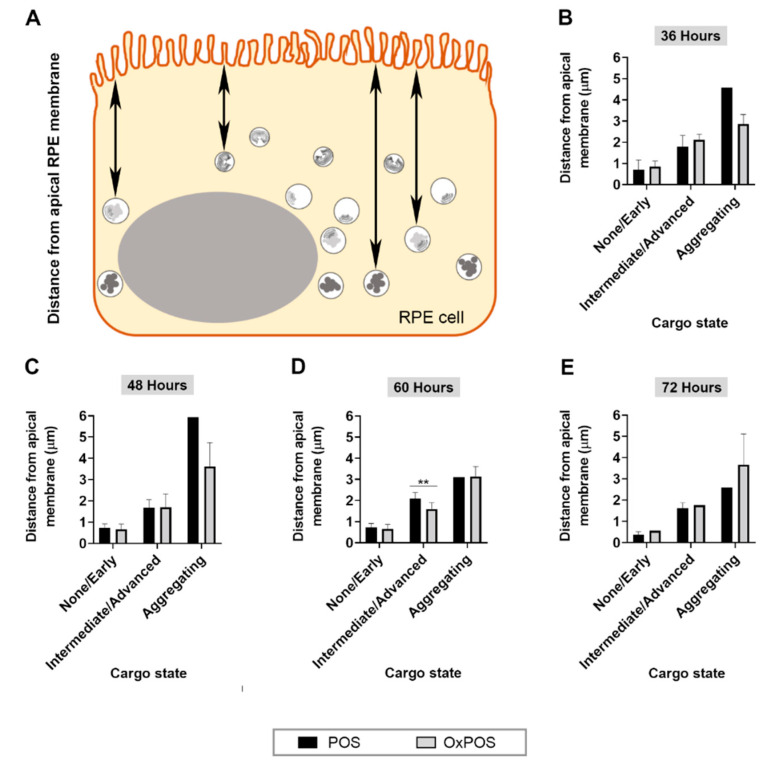
Timelines of POS and OxPOS trafficking as a function of distance from the apical RPE surface. Vesicles containing POS or OxPOS were grouped according to the morphology of their contents: (1) no indication of breakdown or early degradation, (2) intermediate or advanced stages of breakdown or (3) displaying an aggregating phenotype. The distance from the apical RPE cell surface was then measured for each trafficking vesicle. (**A**) Schematic diagram illustrating the method. Blinded electron micrographs were quantified for each group as a function of distance from the apical membrane and shown for the following time points: (**B**) 36 h, (**C**) 48 h, (**D**) 60 h and (**E**) 72 h. Histograms without any error bars indicate where a single vesicle containing presumably endogenous material or OxPOS cargos which were occasionally observed to be degraded were included in the analysis. POS breakdown and degradation of a limited proportion of OxPOS were correlated with increasing time and distance from the apical RPE surface. By contrast, aggregating OxPOS immediately localized to deeper layers of the cell from 36 h onwards. Data from a minimum of *n* = 10 fields/per time point. Statistical comparisons using a two-way ANOVA followed by Tukey’s multiple comparison test, where significant differences between POS vs. OxPOS for each category is indicated as *p* ≤ 0.01 (**).

As processing of cargos in the autophagy and lysosomal pathways are associated with trafficking to deeper cell layers [4], we studied the fate of POS and OxPOS cargos as a function of distance from the apical RPE surface. Cargos were grouped into (1) no obvious breakdown or early indication of degradation, (2) intermediate or advanced stages of breakdown or (3) aggregating, as previously described. Distance was calculated based on where a vertical line originating from the RPE cell surface intersected the apical portion of a trafficking vesicle at mid-point (Figure 6A). At 36 and 48 h, vesicles with POS in progressive stages of degradation were generally associated with increasing distance from the apical surface. A proportion of OxPOS cargos which were degraded also followed a similar pattern, where they were trafficked into deeper layers of the cell (Table S3 and Figure 6B–C). At 60 h following commencement of the feeding assay, differences were recorded between POS vs. OxPOS degradation (Figure 6D). More POS cargos in intermediate/advanced stages of degradation were correlated with increased distance from the apical RPE surface compared to OxPOS in the same category. The final data point at 72 h showed no differences between POS vs. OxPOS correlated with distance from the apical RPE surface (Figure 6E). These findings show that progressive POS degradation alongside the breakdown of a proportion of OxPOS were generally associated with increasingly deeper cell layers, whilst aggregating OxPOS were observed localized to deeper cell layers from 36 h onwards. Furthermore, POS in varying stages of degradation or OxPOS as intracellular aggregates persisted within RPE compartments for at least 3 days after the feeding assay, which was consistent with results from confocal immunofluorescence studies.

### 2.8. OxPOS in RPE Cells Are Correlated with Increased Autofluorescence Levels

**Figure 7:**
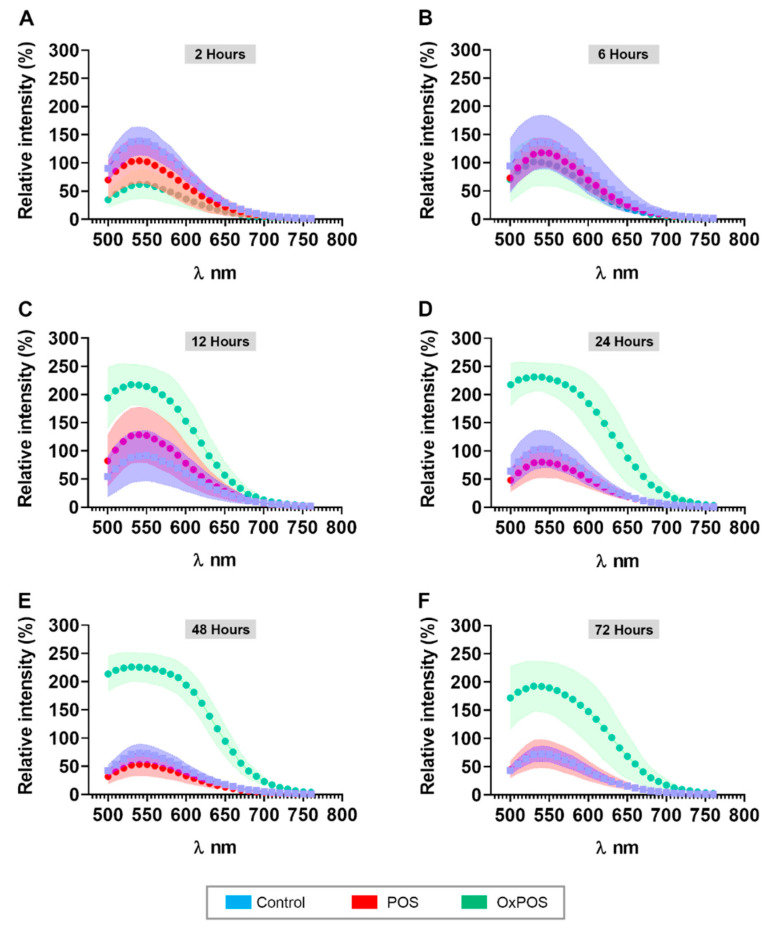
Autofluorescence readouts from POS and OxPOS-fed RPE cells as a function of time. In order to evaluate the spectral signature of RPE cells fed with POS or OxPOS, cultures were excited at 488 nm and lambda scans (emissions) collected at every 10 nm windows. The relative florescence intensity for control (no POS), POS- or OxPOS-fed cultures is shown between 500 and 750 nm for (**A**) 2 h, (**B**) 6 h, (**C**) 12 h, (**D**) 24 h, (**E**) 48 h and (**F**) 72 h, after the feeding assay. Although there were no obvious changes in the autofluorescence readouts at early time points, significant differences emerged between control and POS vs. OxPOS-fed cells after 12 h. Differences between OxPOS vs. POS and controls continued to grow with increasing time, reflecting the contrasting fates between these two cargos. Data from a minimum of *n* = 3 random images/per time point. Statistical studies were performed using a one-way ANOVA followed by Tukey’s multiple comparison tests. For ease of comparing, multiple errors bars between groups are indicated by a color-coded range on either side of the median value (also see Table S4).

As the accumulation of intracellular aggregates are linked with increased autofluorescence in post-mitotic cells [4,30], we quantified autofluorescence levels in RPE cultures fed with POS or OxPOS compared to controls. Following excitation using a 488 nm beam, a λ scan was performed to collect emissions between 500 and 780 nm wavelengths. Values were reported as mean relative fluorescence intensity against emission wavelengths as a function of time (Figure 7A–F). The mean fluorescence intensity in control cultures remained unchanged from 59.88 ± 11.7 at 2 h to 30.85 ± 6.5 at 72 h (Table S4). Interestingly, the mean fluorescence intensity of cultures fed with POS also remained largely consistent from 2 to 72 h, indicating that the proteolytic degradation of these cargos had not significantly contributed to autofluorescence levels, at least in the short term. By contrast, the mean fluorescence intensity of cultures fed with OxPOS increased rapidly from 26.26 ± 11.4 at 2 h to peak from 12 h onwards (104.6 ± 26.5) to 48 h (124.6 ± 20.6), after which they diminished somewhat to 98.85 ± 34.3 by 72 h. Comparison of mean fluorescence intensity values revealed consistent differences between OxPOS vs. POS and control cultures from 12 h onwards (Table S4), indicating that OxPOS contributed to a significant increase in autofluorescence. This can also be visualized by comparing autofluorescence amplitudes between all groups and across different time points, which peak at 535–550 nm wavelengths (Figure 7A–F). From 24 h onwards, OxPOS-fed cultures showed a 3–5-fold increase in autofluorescence compared to POS-fed or control cultures, which persisted until the final 72 h time point.

## 3. Discussion

Elevated proteolytic stress of RPE cells in the aging retina is an important mechanism of early damage which leads to eventual cellular atrophy, a key feature in several blinding conditions. The failure to degrade cargos effectively in a timely manner stems from the dysfunction in one or both of two systems which maintains proteostasis: the ubiquitin proteasome pathway and the autophagy-lysosomal degradation pathway. There is considerable overlap between these, which is not unexpected considering their inter-linked natures [4,6,39,40,41]. Recent evidence suggests that depending on circumstances, cells are able to favor one pathway over the other, including re-purposing or cannibalizing components from one system to boost activities of the other [42,43,44,45]. Analysis of healthy aged as well as diseased/AMD donor tissues indicate a contributory role and/or a direct involvement of these processes with pathology. For instance, the autophagy marker Atg5 was correlated with drusen in healthy aged and AMD eyes [46]. Upregulation of LC3, Atg7 and Atg9 was observed between young vs. older healthy eyes, whilst Atg7 and Atg9A was elevated in age-matched AMD eyes [47]. Moreover, the autophagic receptor SQSTM1/p62 was observed to accumulate in the drusen-rich macular region of donor AMD tissues compared to healthy controls, indicating impaired autophagy in the pathogenesis of AMD [48]. Analysis of donor human retinas demonstrates high levels of lysosomal cathepsin D and acid phosphatase in macula RPE cells relative to RPE in the nasal/mid-zone and peripheral retina [49], indicating the vulnerability of macular RPE. Histological analysis shows aged RPE extruding cytoplasm with active lysosomes into the underlying Bruch’s membrane [50]. RPE cells cultured from donor AMD tissues contain enlarged as well as increased numbers of autophagosomes [51]. A significant body of work has demonstrated that mimicking damage to the mechanisms which regulate proteolysis reproduces characteristics of retinal degeneration [6,11,16,17,18,52,53,54,55,56]. The in vitro model described here also recapitulates salient features observed in pathogenic RPE cells.

Evidence shows that outer segments are sites of significant oxidative modification, which is unsurprising as tissues encompassing the RPE and photoreceptor inner and outer segments were shown to have the highest levels of oxygen consumption compared to adjacent tissues [57]. Studies have demonstrated that following blue light exposure, outer segments are a major source of reactive oxygen species (ROS), triggering an initial over-functioning of photo-transduction and respiratory chain elements in photoreceptors. In a vicious cycle of self-renewal, membrane and protein oxidative damage, proton leakage and uncoupling impairs redox chains, perpetuating damage, causing metabolic dysfunction and photoreceptor apoptosis [58,59,60]. UV-irradiation of isolated POS has been shown to convert regularly arranged membranes into an osmiophilic electron-dense mass which accumulates in cells [13,28,29]. As POS are highly susceptible to oxidative modification, others have harnessed blue light or even exposure to ambient light for their modification [58,61]. Exposure of ARPE-19 cultures to OxPOS leads to upregulation of ERK1/2, a mitogen-activated protein kinase and an upstream activator of the inflammasome-associated transcription factor NF-κB. This results in elevated ROS, increased Bax and Bcl-2 expression, alongside secretion of IL-6, IL-8 and MCP-1 [33]. OxPOS-induced upregulation of VEGF and the aforementioned cytokines appears to be enhanced by the presence of human complement serum [32,33]. An earlier study using ARPE-19 cells also reported the induction of angiogenic cytokines IL-8 and MCP-1 alongside increased ROS following exposure to OxPOS [62]. Continuous exposure of RPE cells to OxPOS for more than 24 h resulted in a significant reduction of complement factor H mRNA [63]. Incubation with OxPOS also led to activation of protein kinase C (PKC) isoforms and PKC-dependent upregulation of the Cdk inhibitor p27kip. Suppression of cell proliferation was observed with multinucleate RPE [64], of which the latter is associated with pathology in the retina. Moreover, OxPOS induced expression of the senescence marker β-galactosidase. Macrophages co-cultured with RPE exposed to OxPOS expressed elevated C1qb, but diminished C3 [65]. There is evidence that OxPOS also target activities of trafficking vesicles. For instance, RPE cells exposed to OxPOS appear to have a diminished capacity to phagocytose POS [66]. Reduced incorporation of a neutral red dye indicates impaired lysosomes in RPE cells fed with OxPOS [32]. Furthermore, OxPOS exposure induced the autophagy marker beclin-1 in ARPE-19 cells [67].

Although OxPOS has been used in previous modeling studies, differences in the beam wavelength, exposure time as well as distance from the UV source may result in some variability across different studies. We used a commercially available device with standardized parameters to generate OxPOS and thus improve consistency, which will be beneficial for future work. Our standardized technique, as well as methods used in earlier studies [28,29,66], resulted in converting regularly arranged POS membranes into an electron-dense mass which accumulate in RPE cells. Standardized OxPOS may also be advantageous for modeling studies, as isolated lipofuscin molecules are likely to introduce variability due to inherent differences in donor biochemistry [13]. Our work and those of others demonstrate that following synthesis, OxPOS undergoes modification, including formation of thiobarbituric acid reactive substrates (TBARS) [13] and carbonyl groups, mimicking features of modifiable macromolecules of in situ RPE cells. However, a notable difference between OxPOS vs. highly purified lipofuscin preparations is the minimal protein content with high levels of oxidative modification in the latter [68], which likely accounts for differences in studies using these respective substrates. In this current study, we compared the trafficking of OxPOS with POS cargos. We observed a consistency in the dynamics of POS trafficking between this investigation and our previously published work [4,27,31]. Most studies typically assay POS trafficking for only 24 h, which shows progressive degradation of cargos with increasing time [18,35]. In a previous investigation, we extended assay times to include 48 h following initial POS feeding [27], and increased it further to 72 h in the current study. Although different RPE cell models are reported to differ greatly in the efficiency and speed of phagocytosis [35], our confocal immunofluorescence as well as ultrastructural data indicate that despite efficient breakdown of POS, some elements of these cargos persist within late compartments of ARPE-19 cells for at least 72 h afterwards. This may have implications for designing future in vitro modeling studies, as the incorporation of extended timescales in experiments could provide additional insights into the etiology of RPE dysfunction. By contrast, the trafficking of OxPOS have not been investigated to this extent before. OxPOS cargos were also trafficked via early and mature lysosomes to LC3b-positive autophagy vesicles. OxPOS were observed co-localized to these compartments from 12 h onwards. Interestingly, OxPOS co-localized puncta appeared larger in size and more conspicuous with a broader cell-wide distribution, and correlated with increasing time following commencement of the pulsed assay, compared to cells fed with POS. Since differences began to emerge between POS vs. OxPOS trafficking from 48 h onwards in the initial screen, we carried out a further series of experiments focused solely on late time points (36–72 h). Combining these results with data from the initial screen generated a powerful dataset, which provided nuanced information on OxPOS trafficking dynamics compared to POS. Whilst POS cargos were sequentially trafficked via LAMP1 and LAMP2 vesicles to LC3b-labeled autophagic vacuoles and degraded, OxPOS cargos were sequestered in these instead. Our observations were consistent in all the confocal immunofluorescence experiments, and subsequently quantified using an automated unbiased algorithm. Generally, a higher frequency of OxPOS cargos was associated with late compartments compared to POS. Once trafficked, OxPOS was retained in these vesicles for at least 72 h, with no evidence of degradation. Measurement of vesicle size confirmed our initial observations in confocal immunofluorescence images, as OxPOS cargos were co-localized with enlarged early and mature lysosomes as well as autophagic vacuoles, with up to a doubling of vesicle size in some instances. In previous work, we reported the swelling of autolysosomes when RPE cells were subject to oxidative stress or following impairment of autophagy [27]. Abnormal swelling of phagosomes/endosomes and autophagosomes have also be demonstrated by others when pathogenic conditions were studied in the RPE [51,69]. Indeed, the presence of swollen endocytic compartments is one of the earliest features in several neuropathies, often preceding the development of clinical symptoms by several decades [70,71]. Our findings add further weight to the likelihood that impaired cargo trafficking and abnormalities in the endosome/phagosome and autophagy-lysosomal pathways is an early feature of neurodegeneration in the retina and brain.

Assessment by TEM revealed ultrastructural insights into the contrasting fates of POS and OxPOS, which supported confocal immunofluorescence microscopy observations. The incorporation of multiple time points can turn even a typically static method such as TEM into one which monitors dynamic intracellular events, including autophagic flux [72]. Whilst the gradual breakdown of POS cargos appear correlated with diminished luminal content and increasing distance from the apical RPE surface, a majority of OxPOS cargos remained visible as conspicuous electron-dense structures, consistent with their morphology reported in previous studies [28,29]. Although there was some indication of degradation, a majority of OxPOS showed no evidence of being processed by proteolytic mechanisms. Use of immunogold TEM approaches in future studies may allow estimation of OxPOS breakdown as a proportion of stable OxPOS aggregates. The latter remained as stable, largely unperturbed intracellular aggregates for at least 72 h after the feeding assay, and generally co-localized to deeper cell layers from 36 h onwards. Next, we assessed autofluorescence, a feature of RPE cells which is attributed to these intracellular macromolecules [30]. Although naturally formed lipofuscins differ in their complexity from artificially synthesized OxPOS, previous studies have noted some overlap, including autofluorescent properties, which were recorded following OxPOS internalization by RPE cells. OxPOS fluorescence was measured in previous studies using a 450–490 nm excitation filter containing a dichromatic beam-splitter with 50% reflection at 510 nm and a barrier filter at 520 nm, with an additional 550 nm filter to eliminate non-lipofuscin autofluorescence [28,29,66]. Other studies employed a plate reader detecting signals at 490 nm [32]. We used the capabilities of a powerful research-grade laboratory confocal microscope to recapitulate parameters of the Heidelberg Spectralis, which excites with a 488 nm beam to measure fundus autofluorescence (FAF) in patients. Use of FAF, of which blue light excitation is the most widely used, is an accepted clinical end-point and a gold standard for assessing RPE changes in patients [73]. For instance, FAF reveals areas of RPE loss as dark patches in retinas of geographic atrophy AMD patients. The method is also used to assess abnormal autofluorescence levels in the junctional zone, between atrophic and the normal retina, as this often precedes cell death [74]. Longitudinal assessments indicate that enlargement of areas with FAF accumulation surrounding atrophy is strongly correlated with disease progression over time [75]. Analysis of donor tissues show diminishing autofluorescence correlated with reduced autofluorescent organelle content in RPE cells [76], supporting more recent clinical quantitative autofluorescent imaging studies, which suggests that lipofuscin and its autofluorescence signal is stable or even declines in AMD [77,78]. The comparison of autofluorescence signals in living eyes and donor human tissues [79] vs. signals from cultured RPE cells with OxPOS revealed some similarities as well as differences. To our knowledge, our report is the first instance where this non-invasive method of evaluating RPE health in patients has been directly applied in obtaining autofluorescence readouts of cultured RPE in the laboratory. Although both readouts showed a broad band of emissions between 500 and 750 nm, optimal excitation was achieved at 510 nm in native tissues, whilst peak excitation was recorded between 535 and 550 nm in OxPOS-treated RPE cultures. These differences may be attributed to several factors as autofluorescence readouts from a simple in vitro system cannot reproduce like-for-like signals from multiple cell-types in native tissues. Comparison of autofluorescence values between cultures exposed to different treatments indicate that a single POS pulse did not appreciably contribute to increased autofluorescence over baseline values, even in RPE cells that had been in culture for several months. This observation was consistent with an earlier study showing a similar outcome [28]. However, another study reported POS contributing to autofluorescence levels in cultured RPE cells [32]. By contrast, a single OxPOS pulse resulted in a significant increase in autofluorescence, as much as 3–5-fold over POS-fed or control cultures, suggesting that our model can recapitulate an important biomarker of RPE health that is clinically linked to retinopathy.

**Figure 8:**
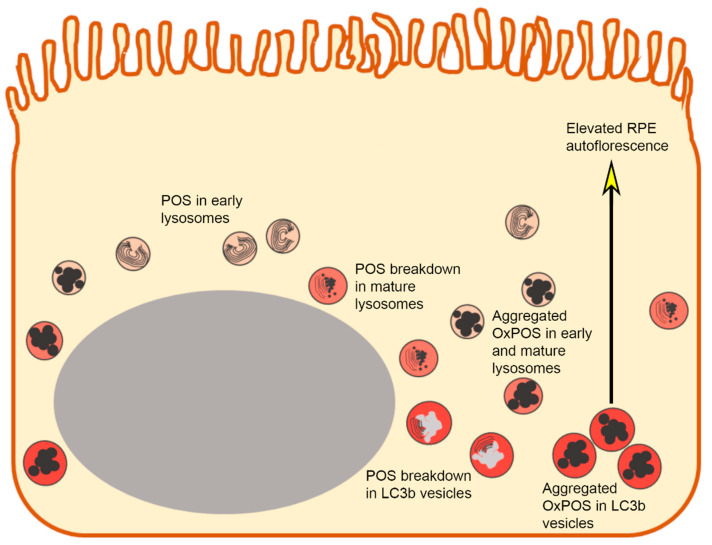
Summary diagram showing an in vitro RPE cell model recapitulating pathogenic features following exposure to OxPOS. (**a**) The accumulation of intracellular macromolecules, (**b**) oxidative stress and (**c**) damaged intracellular cargo trafficking and autophagy.

In summary, the findings described herein alongside work published by others demonstrates that this in vitro RPE cell model is a useful tool to study (a) the effects of aggregating intracellular macromolecules, (b) effects of oxidative stress, as well as (c) damage to cargo trafficking/processing, which are associated with RPE dysfunction. The key findings from this study relate to points (a) and (c), which are summarized in Figure 8, whilst OxPOS-induced oxidative and angiogenic effects have been described by others.

The accumulation of pathogenic intracellular macromolecules is also a feature of Alzheimer’s disease. Hence, studies using this model could have implications for understanding and developing treatments for neuropathies [80]. Since the intake of a high-fat diet, which is also associated with elevated oxidative stress as well as impaired autophagy, converges on protein aggregation [27,81], this model could be further exploited to elucidate how these pathways are linked. The versatility of in vitro cell models of this kind, in particular the capability to study molecular events at single-cell resolution, has already led to unravelling novel disease-causing mechanisms that underlie irreversible sight-loss. Exploitation of such pre-clinical in vitro models also brings substantial benefits, as many drug-discovery pathways seek to avoid the use of unsatisfactory animal models of retinal degeneration where possible [82] and transition directly to clinical trials.

## 4. Materials and Methods

### 4.1. Cell Culture

The human cell line ARPE-19 [83] was obtained from the American Tissue Culture Collection (ATCC, Manassas, VA, USA) and maintained in a 37 °C humidified incubator with 5% CO_2_ and 95% air. Cells were cultured in Dulbecco’s modified Eagle’s medium (DMEM) with 4.5 g/L L-D glucose, L-glutamine and pyruvate (Life Technologies, Warrington, UK), supplemented with 1% heat-inactivated fetal calf serum (Sigma Aldrich, Poole, Dorset, UK) and 1% penicillin-streptomycin stock solution (10,000 units/mL penicillin, 10 mg/mL streptomycin in 0.85% saline (Sigma Aldrich, UK)). Cells cultured in a T-25 flask were maintained in a 5 mL volume of medium, with a complete media change performed every 3–4 days. Cells were seeded at a density of 1.25 × 10^4^/well on 12 mm diameter 0.4 µm pore PET polyester transwell inserts (Sigma Aldrich, Poole, Dorset, UK), which were pre-coated with 50 µg/mL fibronectin (Sigma-Aldrich, Poole, Dorset, UK), and cultured for up to 4 months prior to experiments. Cells were maintained in 0.5 and 2 mL media volumes in apical and basal chambers, respectively. A complete medium change in the apical compartment and a 20% (v) change in the basal compartment was performed twice a week, as described before [26,27].

### 4.2. Photoreceptor Outer Segment (POS) Pulse Assay

Retinas were isolated from porcine eyes and pooled in KCl buffer (0.3 M KCl, 10 mM HEPES, 0.5 mM CaCl_2_, 2 mM MgCl_2_) in 48% sucrose solutions. Retinas were homogenized by gentle shaking for 2 min. The solution was then centrifuged at 5000× *g* for 5 min before the supernatant was passed through a sterile gauze into fresh centrifuge tubes and diluted with KCl buffer without sucrose. This preparation was subsequently centrifuged at 4000× *g* for 7 min, after which the pellet was washed 3 times in PBS through centrifugation at 4000× *g* for a further 7 min. POS were resuspended in 20 mM phosphate buffer (pH 7.2) with 10% sucrose and 5 µM taurine. Half of the POS preparation was UV-cross-linked by exposure to a 254 nm beam for 3 h, which delivered 120,000 µJ (UVP CX-2000 UV cross linker, Fisher Scientific, Loughborough, UK). The FITC conjugate (ThermoFisher, Loughborough, UK) was added to POS and OxPOS preparations and the solution was left on a rotating plate for 1 h in the dark to allow for covalent attachment of the fluorescent tag. The POS-FITC solution was then centrifuged at 3000× *g* for 4 min at 20 °C, suspended in DMEM with 2.5% sucrose, aliquoted and stored at −80 °C. Isolated POS were quantified using a BCA assay (Pierce, ThermoFisher, Loughborough, UK), in which proteins were measured against standards between 20 and 2000 µg/mL via absorption at 562 nm (Infinite F200 Pro, Tecan, Männedorf, Switzerland). RPE monolayers were chilled to 17 °C for 30 min and pulsed with POS or OxPOS. We used 4 µg/cm^2^ of POS-FITC [21] in a synchronized pulsed assay where cultures were incubated at 17 °C for a further 30 min to maximize binding whilst minimizing cargo internalization [84]. The medium was aspirated to remove unbound POS, replaced with fresh pre-warmed medium and cultures returned to a humidified incubator at 37 °C with 5% CO_2_. Cultures were fixed at time points that are specified below. Data were from a minimum of three independent experiments with at least three samples per measurement.

### 4.3. Determination of Protein Carbonyl Content

Samples were dissolved in ddH_2_O and centrifuged at 16,000× *g* for 1 min to remove any insoluble material. Samples were diluted to obtain a concentration of 10 mg/mL protein. A sample volume of 100 µL was used for the assay. 100 µL 2,4-dinitrophenylhydrazine (DNPH) (Abcam, Cambridge, UK) was added to each sample, vortexed and incubated for 10 min at room temperature. 30 µL of 87% trichloroacetic acid (TCA) (Abcam, Cambridge, UK) was subsequently added to each sample, vortexed and left on ice for 5 min. Samples were then centrifuged at 16,000× *g* for 2 min and the supernatant was discarded. 500 µL of ice-cold acetone (Sigma Aldrich, Poole, Dorset, UK) was added to each tube and placed in a sonicating bath for 30 s. Samples were chilled at −20 °C for 5 min before centrifugation at 16,000× *g* for 2 min and the acetone was removed. The acetone step was repeated to remove any unbound DNPH before 200 µL 6M Guanidine solution (Abcam, Cambridge, UK) was added and the samples were sonicated briefly. 100 µL of each sample was added to a 96-well plate and the optical density (OD) was measured at 375 nm in a spectrophotometer. Data were from three independent experiments with at least three samples per measurement. A BCA was performed to determine the quantity of total proteins and values presented as nanomolar carbonyl content per mg of total protein.

### 4.4. Confocal Immunofluorescence Microscopy and Co-Localization Studies

Cultures were fixed in 4% formaldehyde (in 0.1 M phosphate-buffered saline (PBS)) for 20 min at 4 °C, permeabilized in 0.1% Triton-X 100 for 30 min and blocked in PBS containing 1% BSA and 0.1% Tween for a further 30 min. Cells were then incubated with the primary antibody diluted in the same solution at 4 °C overnight. Cultures were probed with the following antibodies: rabbit anti-LAMP1 (AB_775978; 1:1000), rabbit anti-LAMP2 (AB_755981; 1:1000) and rabbit anti-LC3b (AB_881433; 1:200). Following removal of unbound antibodies by washing, cells were incubated for 1 h at room temperature with an Alexa Fluor-conjugated secondary antibody (AB_2534116), which was diluted to 1:100 in PBS-Tween with BSA. In all studies, 1 µg/mL of 4′,6′-diamino-2-phenylindole (DAPI: D9542, Sigma Aldrich, Poole, Dorset, UK) was used to visualize cell nuclei. Samples were mounted between two glass coverslips using Mowiol, and images were acquired using a Leica SP8 (Leica Microsystems, Milton Keynes, UK) confocal laser scanning microscope. Co-localization was visually assessed using a maximum projection of confocal z-stacks throughout the full thickness of the specimen (system optimized: which takes 0.33 µm thick optical slices) which are projected as a single image. Data were from at least three independent experiments with three or more separate culture dishes per measurement. Quantification of POS-FITC and OxPOS-FITC in various intracellular compartments was carried out using Volocity software (Perkin Elmer, Beaconsfield, UK), which uses an automated unbiased algorithm described by Costes et al. [36]. Co-localization values were plotted for each compartment as a function of time. The size of intracellular vesicles was measured by Fiji software (NIH, Bethesda, MD, USA) using single-plane images from the middle of confocal z-stacks at 200x magnification, as described before [27,85] (*n* = 5, with separate measurements for each compartment/time point/group). Size measurements were carried out using only the red channel (vesicle marker). POS- or OxPOS-containing vesicles were pre-identified and the green channel was switched off to remove any fluorescence flare interfering with measurements. Values were recorded for vesicles containing POS or OxPOS as well as for vesicles without these cargos.

### 4.5. Transmission Electron Microscopy (TEM)

ARPE-19 monolayers pulsed with POS or OxPOS were fixed with primary fixative comprising 3% glutaraldehyde, 4% formaldehyde in 0.1 M PIPES buffer (pH 7.2) for a minimum of 1 h. Specimens were then rinsed in 0.1 M PIPES buffer, post-fixed in 1% buffered osmium tetroxide for 1 h, rinsed in buffer and block-stained in 2% aqueous uranyl acetate (20 min). Samples were then dehydrated in an ethanol gradient (30%, 50%, 70%, 95%) for 10 min each and twice in absolute ethanol for 20 min. The link reagent acetonitrile was then applied for 10 min, after which samples were incubated overnight in a 1:1 ratio of acetonitrile to Spurr resin. The following day, cells were incubated in fresh Spurr resin for 6 h before being embedded and polymerized in Spurr resin (Agar Scientific, Stanstead, UK) at 60 °C for 24 h. Silver/gold ultrathin sections were cut on a Reichert Ultracut E ultramicrotome (Leica Microsystems, Milton Keynes, UK), collected on 200 mesh copper grids and stained with Reynolds lead stain. Sections were viewed using a Hitachi HT7700 (Hitachi High-Tech, Tokyo, Japan) transmission electron microscope. Fiji (NIH, Bethesda, MD, USA) analysis was carried out using blinded micrographs, where *n* ≤ 10 images were collected for each time point. Distance from the apical membrane was measured at 90° from the cell surface to the closest point of the vesicle. All complete vesicles with intersecting perpendicular apical membrane were included in the study. The contents of trafficking vesicles were analyzed by drawing a ROI around each vesicle and quantifying the mean luminal greyscale value. Mean values were calculated for each treatment group per time point. Data were from three independent experiments with at least three samples per measurement.

### 4.6. Autofluorescence Measurements in Cultured RPE Cells

Control cultures (without exposure to either POS or OxPOS), as well as sister cultures exposed to POS or OxPOS, were fixed at 2, 6, 12, 24, 48 and 72 h following commencement of the pulsed feeding assay. These were long-term cultures of ARPE-19 cells in transwell inserts, which were then fixed with 4% paraformaldehyde PFA for 10 min, stained for 4′,6-diamidino-2-phenylindole (DAPI) and prepared for confocal microscopy, as described before. Transwell culture samples were excited with a 488 nm wavelength laser and cell autofluorescence was measured using lambda (λ) scans along a 500–780 nm window, using a step-size of 10 nm and a detection bandwidth of 20 nm. The z-plane for λ scanning was selected when the DAPI channel was in focus, to ensure that an equivalent focal plane was used across all transwells. Images were analyzed using Leica AS Lite software (Leica Microsystems, UK). ROIs were drawn around 5 cells per image, and a λ scan profile (autofluorescence signal strength across the detection wavelength range) was generated and plotted. Results were obtained from three independent experiments with at least three samples per measurement. The data was exported into a Microsoft Excel spreadsheet to obtain mean and standard deviation (SD) values of autofluorescence intensity for each λ scan measurement window for each sample group and time point (*n* = 3 per group/time point).

### 4.7. Statistical Analysis

Statistical comparisons were performed using GraphPad prism Software (GraphPad, Prism 8.2. 1, San Diego, CA, USA). The statistical differences were determined using an unpaired Student’s t-test or a one-way analysis of variance (ANOVA). Data is expressed as a mean value ± standard deviation (SD), where significance is denoted as follows: *p* ≤ 0.05 (*), *p* ≤ 0.01 (**), *p* ≤ 0.001 (***) and *p* ≤ 0.0001 (****).

## Supplementary Materials

Supplementary materials can be found at https://www.mdpi.com/1422-0067/21/18/6647/s1.
