# Supplementary material for: An In-Vitro Cell Model of Intracellular Protein Aggregation Provides Insights into RPE Stress Associated with Retinopathy
Source: Int J Mol Sci. 2020 Sep 11;21(18):6647. doi: 10.3390/ijms21186647 (PMC7555953; doi:10.3390/ijms21186647)
Supplement: Supplementary file 1 [file ijms-21-06647-s001.pdf]

Supplementary information

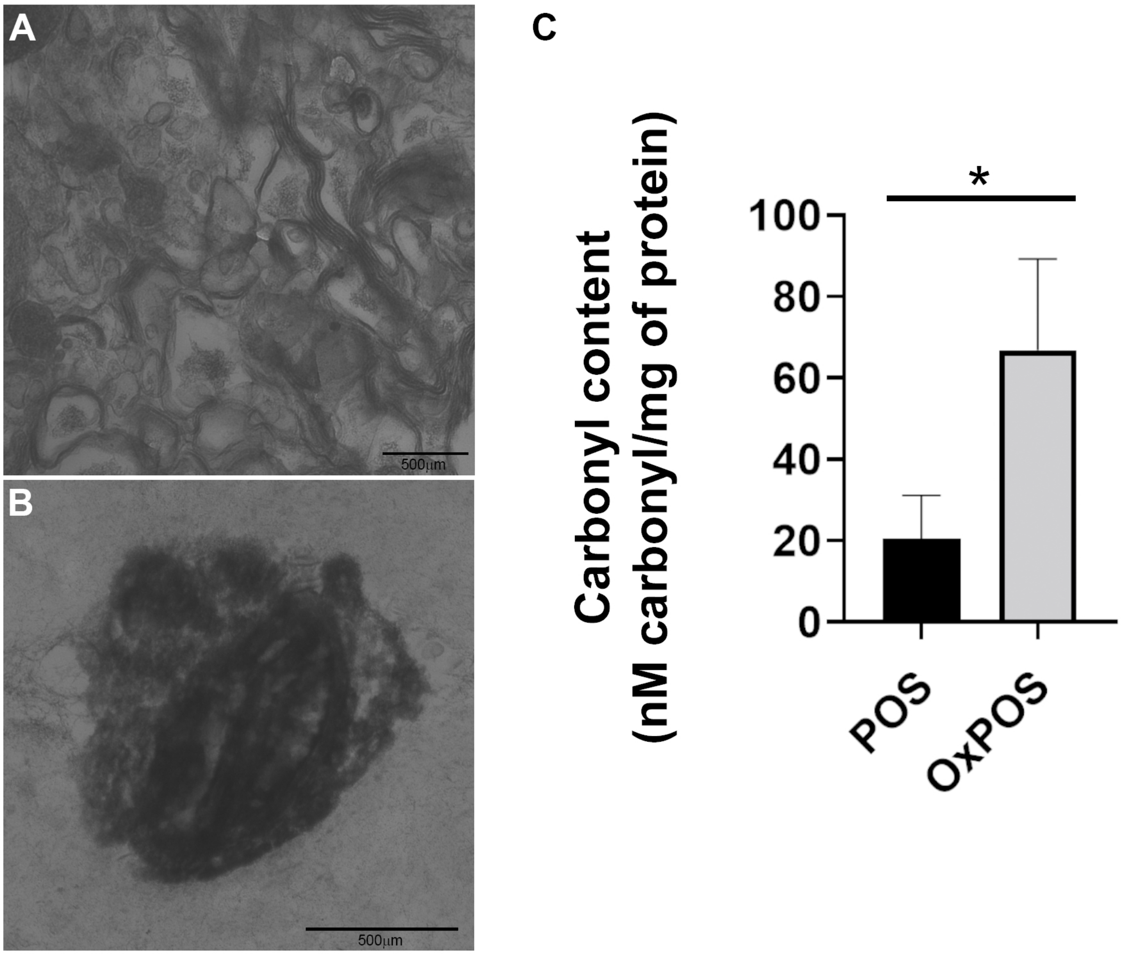

Figure S1 Ultrastructural and biochemical characterization of photoreceptor outer segments and modifications by UV-irradiation. Photoreceptor outer segments (POS) and modified POS (OxPOS) preparations were analyzed *in-vitro* by transmission electron microscopy. Representative electron micrographs show (A) the regular organization of outer segment membranes, which (B) when oxidatively modified coalesced into an electron-dense aggregate. Scale bars correspond to 500nm. (C) The carbonyl content of POS and OxPOS preparations were assessed i*n-vitro*, prior to incubation with cultured RPE cells. An assay that tags DNP hydrazones to carbonyl groups was carried out, the readouts of which can be quantified by a spectrophotometer set at 375nm. Data from n=3 independent experiments. Statistical comparisons using a student's t-test, where a significant difference between POS vs. OxPOS is indicated as $p < 0.05$ (*).

Supplementary Table 1

	Data from initial screen											
	Extent of co-localization											
	12 Hours				18 Hours				24 Hours			
	POS		OxPOS		POS		OxPOS		POS		OxPOS	
	Mean	SD	Mean	SD	Mean	SD	Mean	SD	Mean	SD	Mean	SD
Lamp 1	0.61	0.20	0.82	0.15	0.78	0.13	0.84	0.15	0.72	0.17	0.87	0.12
Lamp 2	0.81	0.11	0.87	0.12	0.86	0.10	0.89	0.08	0.88	0.11	0.91	0.11
LC3B	0.77	0.20	0.91	0.11	0.73	0.18	0.90	0.09	0.78	0.14	0.92	0.07

	Data from initial screen combined with data from experiments focused on late time points															
	Extent of co-localization															
	36 Hours				48 Hours				60 Hours				72 Hours			
	POS		OxPOS		POS		OxPOS		POS		OxPOS		POS		OxPOS	
	Mean	SD	Mean	SD	Mean	SD	Mean	SD	Mean	SD	Mean	SD	Mean	SD	Mean	SD
Lamp 1	0.66	0.20	0.81	0.13	0.73	0.17	0.88	0.12	0.50	0.24	0.89	0.10	0.39	0.19	0.93	0.05
Lamp 2	0.76	0.14	0.84	0.13	0.73	0.16	0.88	0.11	0.55	0.23	0.89	0.09	0.30	0.17	0.92	0.06
LC3B	0.76	0.17	0.81	0.15	0.81	0.12	0.88	0.14	0.75	0.19	0.94	0.06	0.62	0.23	0.92	0.07

Note: Extent of co-localization where 1.0 = 100%. SD: Standard deviation

Supplementary Table 2

Sizes of compartments (μm)

36 Hrs

Lamp 1	No POS	POS	No OxPOS	OxPOS
No POS	Mean = 0.7996 ± 0.18	p = 0.7412	p = 0.8375	p = 0.8898
POS	p = 0.7412	Mean =0.8707 ± 0.26	p = 0.2549	p = 0.9909
No OxPOS	p = 0.8375	p = 0.2549	Mean = 0.7355 ± 0.11	p =0.4089
OxPOS	p =0.8898	p =0.9909	p = 0.4089	Mean = 0.8490 ± 0.19

Lamp 2	No POS	POS	No OxPOS	OxPOS
No POS	Mean = 0.7961 ± 0.17	p = 0.7692	p = 0.9997	p = 0.9870
POS	p = 0.7692	Mean = 0.8712 ± 0.23	p = 0.8216	p = 0.9221
No OxPOS	p = 0.9997	p = 0.8216	Mean = 0.8037 ± 0.20	p = 0.9953
OxPOS	p = 0.9870	p = 0.9221	p = 0.9953	Mean = 0.8222 ± 0.21

LC3b	No POS	POS	No OxPOS	OxPOS
No POS	Mean = 0.6746 ± 0.13	p = 0.9135	p = 0.9795	p = 0.9947
POS	p = 0.9135	Mean = 0.7237 ± 0.14	p = 0.7223	p = 0.9768
No OxPOS	p = 0.9795	p = 0.7223	Mean = 0.6452 ± 0.11	p = 0.9197
OxPOS	p = 0.9947	p = 0.9768	p = 0.9197	Mean = 0.6930 ± 0.12

48 Hrs

Lamp 1	No POS	POS	No OxPOS	OxPOS
No POS	Mean = 0.7225 ± 0.11	p = 0.9503	p = 0.9911	p = 0.7481
POS	p = 0.9503	Mean = 0.7616 ± 0.16	p = 0.8405	p = 0.9660
No OxPOS	p = 0.9911	p = 0.8405	Mean = 0.7010 ± 0.14	p = 0.5667
OxPOS	p = 0.7481	p = 0.9660	p = 0.5667	Mean = 0.7958 ± 0.20

Lamp 2	No POS	POS	No OxPOS	OxPOS
No POS	Mean = 0.6847 ± 0.13	p = 0.6886	p = 0.8902	p = 0.5965
POS	p = 0.6886	Mean = 0.7704 ± 0.12	p = 0.9804	p = 0.9989
No OxPOS	p = 0.8902	p = 0.9804	Mean = 0.7405 ± 0.17	p = 0.9510
OxPOS	p = 0.5965	p = 0.9989	p = 0.9510	Mean = 0.7819 ± 0.16

LC3b	No POS	POS	No OxPOS	OxPOS
No POS	Mean = 0.6502 ± 0.13	p = 0.5538	p = 0.9075	p = 0.0543
POS	p = 0.5538	Mean = 0.7513 ± 0.16	p = 0.9057	p = 0.6144
No OxPOS	p = 0.9075	p = 0.9057	Mean = 0.7006 ± 0.13	p = 0.2339
OxPOS	p = 0.0543	p = 0.6144	p = 0.2339	Mean = 0.8429 ± 0.28

60 Hrs

Lamp 1	No POS	POS	No OxPOS	OxPOS
No POS	Mean = 0.7182 ± 0.11	p = 0.6411	p = 0.9993	p = 0.0021
POS	p = 0.6411	Mean = 0.8044 ± 0.16	p = 0.7172	p = 0.0689
No OxPOS	p = 0.9993	p = 0.7172	Mean = 0.7273 ± 0.12	p = 0.0032
OxPOS	p = 0.0021	p = 0.0689	p = 0.0032	Mean = 0.9853 ± 0.15

Lamp 2	No POS	POS	No OxPOS	OxPOS
No POS	Mean = 0.6636 ± 0.10	p = 0.9175	p = 0.7746	p < 0.0001
POS	p = 0.9175	Mean = 0.7136 ± 0.13	p = 0.9894	p < 0.0001
No OxPOS	p = 0.7746	p = 0.9894	Mean = 0.7379 ± 0.14	p < 0.0001
OxPOS	p < 0.0001	p < 0.0001	P < 0.0001	Mean = 1.143 ± 0.12

LC3b	No POS	POS	No OxPOS	OxPOS
No POS	Mean = 0.6525 ± 0.12	p =0.7502	p = 0.3752	p < 0.0001
POS	p = 0.7502	Mean = 0.7274 ± 0.11	p = 0.9276	p < 0.0001
No OxPOS	p = 0.3752	p = 0.9276	Mean = 0.7734 ± 0.15	p = 0.0002
OxPOS	p <0.0001	p <0.0001	p = 0.0002	Mean = 1.009 ± 0.17

72 Hrs

Lamp 1	No POS	POS	No OxPOS	OxPOS
No POS	Mean = 0.6571 ± 0.12	p = 0.6386	p = 0.8228	p < 0.0001
POS	p = 0.6386	Mean = 0.7436 ± 0.12	p = 0.9888	p < 0.0001
No OxPOS	p = 0.8228	p = 0.9888	Mean = 0.7204 ± 0.13	p < 0.0001
OxPOS	p < 0.0001	p <0.0001	p < 0.0001	Mean = 1.255 ± 0.20

Lamp 2	No POS	POS	No OxPOS	OxPOS
No POS	Mean = 0.6069 ± 0.10	p = 0.4176	p = 0.9369	p < 0.0001
POS	p = 0.4176	Mean = 0.7265 ± 0.15	p = 0.7927	p < 0.0001
No OxPOS	p = 0.9369	p = 0.7927	Mean = 0.6548 ± 0.12	p < 0.0001
OxPOS	p < 0.0001	p < 0.0001	p < 0.0001	Mean = 1.336 ± 0.24

LC3b	No POS	POS	No OxPOS	OxPOS
No POS	Mean = 0.6130 ± 0.11	p = 0.2665	p = 0.8728	p < 0.0001
POS	p = 0.2655	Mean = 0.7498 ± 0.11	p = 0.7107	p < 0.0001
No OxPOS	p = 0.8728	p = 0.7107	Mean = 0.6698 ± 0.14	p < 0.0001
OxPOS	p <0.0001	p < 0.0001	p < 0.0001	Mean = 1.355 ± 0.28

Note: SD = Standard deviation

Supplementary Table 3

Distance from apical the RPE membrane

36 Hrs	None/Early		Intermediate/ Advacned		Aggregating	
	POS	OxPOS	POS	OxPOS	POS	OxPOS
POS	Mean = 0.70 ± 0.47	p= 0.9993	Mean = 1.8 ± 0.53	p= 0.5584	Mean = 4.57 ± 0	
OxPOS	p= 0.9993	Mean = 0.85 ± 0.26	p= 0.5584	Mean = 2.12 ± 0.26		Mean = 2.86 ± 0.44

48 Hrs	None/Early		Intermediate/ Advacned		Aggregating	
	POS	OxPOS	POS	OxPOS	POS	OxPOS
POS	Mean = 0.72 ± 0.19	p= 0.9999	Mean = 1.7 ± 0.38	p= 0.9999	Mean = 5.93 ± 0	
OxPOS	p= 0.9999	Mean = 0.66 ± 0.25	p= 0.9999	Mean = 1.7 ± 0.64		Mean = 3.61 ± 1.13

60 Hrs	None/Early		Intermediate/ Advacned		Aggregating	
	POS	OxPOS	POS	OxPOS	POS	OxPOS
POS	Mean = 0.726 ± 0.19	p= 0.9948	Mean = 2.1 ± 0.30	p = 0.0055	Mean = 3.12 ± 0	
OxPOS	p= 0.9948	Mean = 0.66 ± 0.21	p= 0.0055	Mean = 1.6 ± 0.30		Mean = 3.13 ± 0.48

72Hrs	None/Early		Intermediate/ Advacned		Aggregating	
	POS	OxPOS	POS	OxPOS	POS	OxPOS
POS	Mean = 0.38 ± 0.13	p= 0.9828	Mean = 1.62 ± 0.26		Mean = 2.6 ± 0	
OxPOS	p= 0.9828	Mean = 0.56 ± 0		Mean = 1.76 ± 0		Mean = 3.66 ± 1.45

Note: Only mean values are shown were data points in at least one group contained n=1. SD = Standard deviation

Supplementary Table 4

Autofluorescence data

2 Hours

	No POS (control)	POS	OxPOS
No POS (control)	Mean = 59.66 ± 11.7	p = 0.3302	p = 0.0075
POS	p = 0.3302	Mean = 44.24 ± 21.4	p = 0.2238
OxPOS	p = 0.0075	p = 0.2238	Mean = 26.26 ± 11.04

6 Hours

	No POS (control)	POS	OxPOS
No POS (control)	Mean = 61.95 ± 27.7	p = 0.6215	p = 0.8003
POS	p = 0.6215	Mean = 50.59 ± 13.5	p = 0.2649
OxPOS	p = 0.8003	p = 0.2649	Mean = 42.83 ± 17.7

12 Hours

	No POS (control)	POS	OxPOS
No POS (control)	Mean = 41.73 ± 22.5	p = 0.6675	p = 0.0008
POS	p = 0.6675	Mean = 55.89 ± 24.8	p = 0.0114
OxPOS	p = 0.0008	p = 0.0114	Mean = 104.6 ± 26.5

24 Hours

	No POS (control)	POS	OxPOS
No POS (control)	Mean = 43.24 ± 14.8	p = 0.875	p < 0.0001
POS	p = 0.875	Mean = 35.24 ± 12.2	p < 0.0001
OxPOS	p < 0.0001	p < 0.0001	Mean = 122.7 ± 29.8

48 Hours

	No POS (control)	POS	OxPOS
No POS (control)	Mean = 31.87 ± 7.5	p = 0.8446	p < 0.0001
POS	p = 0.8446	Mean = 23.46 ± 9.1	p < 0.0001
OxPOS	p < 0.0001	p < 0.0001	Mean = 124.6 ± 20.6

72 Hours

	No POS (control)	POS	OxPOS
No POS (control)	Mean = 30.85 ± 6.5	p = 0.9994	p < 0.0001
POS	p = 0.9994	Mean = 31.28 ± 11.6	p < 0.0001
OxPOS	p < 0.0001	p < 0.0001	Mean = 98.85 ± 34.3

Note: SD = Standard deviation
